# Using novel acoustic and visual mapping tools to predict the small-scale spatial distribution of live biogenic reef framework in cold-water coral habitats

**DOI:** 10.1007/s00338-016-1519-8

**Published:** 2016-12-05

**Authors:** L. H. De Clippele, J. Gafeira, K. Robert, S. Hennige, M. S. Lavaleye, G. C. A. Duineveld, V. A. I. Huvenne, J. M. Roberts

**Affiliations:** 1 Centre for Marine Biodiversity and Biotechnology, School of Energy, Geoscience, Infrastructure and Society, Heriot-Watt University, Edinburgh, EH14 4AS UK; 2 British Geological Survey, Lyell Centre, Research Ave S, Edinburgh, EH14 4AP UK; 3 Marine Geoscience, National Oceanographic Centre, University of Southampton Waterfront Campus, European Way, Southampton, SO14 3ZH UK; 4 NIOZ Royal Netherlands Institute for Sea Research, Department of Ocean Systems Sciences, and Utrecht University, PO Box 59, 1790 AB Den Burg, Texel, The Netherlands; 5 University of Edinburgh, Grant Institute, James Hutton Road, Edinburgh, EH9 3FE UK

**Keywords:** Microbathymetry, BGS seabed mapping toolbox, *Lophelia pertusa*, Random forest classification, Predictive modelling

## Abstract

Cold-water corals form substantial biogenic habitats on continental shelves and in deep-sea areas with topographic highs, such as banks and seamounts. In the Atlantic, many reef and mound complexes are engineered by *Lophelia pertusa*, the dominant framework-forming coral. In this study, a variety of mapping approaches were used at a range of scales to map the distribution of both cold-water coral habitats and individual coral colonies at the Mingulay Reef Complex (west Scotland). The new ArcGIS-based British Geological Survey (BGS) seabed mapping toolbox semi-automatically delineated over 500 *Lophelia* reef ‘mini-mounds’ from bathymetry data with 2-m resolution. The morphometric and acoustic characteristics of the mini-mounds were also automatically quantified and captured using this toolbox. Coral presence data were derived from high-definition remotely operated vehicle (ROV) records and high-resolution microbathymetry collected by a ROV-mounted multibeam echosounder. With a resolution of 0.35 × 0.35 m, the microbathymetry covers 0.6 km^2^ in the centre of the study area and allowed identification of individual live coral colonies in acoustic data for the first time. Maximum water depth, maximum rugosity, mean rugosity, bathymetric positioning index and maximum current speed were identified as the environmental variables that contributed most to the prediction of live coral presence. These variables were used to create a predictive map of the likelihood of presence of live cold-water coral colonies in the area of the Mingulay Reef Complex covered by the 2-m resolution data set. Predictive maps of live corals across the reef will be especially valuable for future long-term monitoring surveys, including those needed to understand the impacts of global climate change. This is the first study using the newly developed BGS seabed mapping toolbox and an ROV-based microbathymetric grid to explore the environmental variables that control coral growth on cold-water coral reefs.

## Introduction

Cold-water coral reefs are long-living, slow-growing hot spots of biodiversity threatened by fishing activities and climate change (Freiwald et al. 2004; Costello et al. 2005; Roberts et al. 2009a). Improvement in our understanding of the distribution of these species and their habitats is necessary to protect these ecosystems efficiently. Over recent decades, *Lophelia pertusa* has been the most studied cold-water coral in the world (Costello et al. 2005; Roberts et al. 2009a). *Lophelia pertusa* can be defined as an ‘ecosystem engineer’ (Jones et al. 1994) as reefs formed by this coral alter the local hydrodynamics and sediment deposition and provide a complex three-dimensional habitat for many other species including communities of suspension-feeding invertebrates such as sponges and other corals (Jensen and Frederiksen 1992; Henry and Roberts 2007; Buhl-Mortensen et al. 2010).

*Lophelia pertusa* reefs form under specific environmental conditions that are not yet fully understood (Davies et al. 2008; Howell et al. 2011) but which are known to be controlled by a complex interplay of physical, chemical and biological factors (Thiem et al. 2006). *Lophelia pertusa* is a widely distributed species that requires a hard substrate such as boulders, pebbles or shells for initial attachment (Wilson 1979; Hovland et al. 2005). It is preferentially associated with areas of accelerated near-bed currents which are often found on sloping topographies and topographic highs (Genin et al. 1986; Frederiksen et al. 1992). This is because the living coral polyps feed on particles suspended in the water, and the encounter rate of food increases with higher current speeds (Frederiksen et al. 1992; Mortensen 2001; Thiem et al. 2006). In particular, phytoplankton is predominantly captured at higher flow velocities of 0.5 cm s^−1^, but zooplankton is successfully captured at lower flow velocities of 0.2 cm s^−1^. *Lophelia pertusa* can occur at depths of 39–3380 m. The corals are associated with temperatures between 4 and 13 °C (Roberts et al. 2009a) and an oceanic salinity around 35 on the practical scale (Freiwald et al. 2004).

**Fig. 1:**
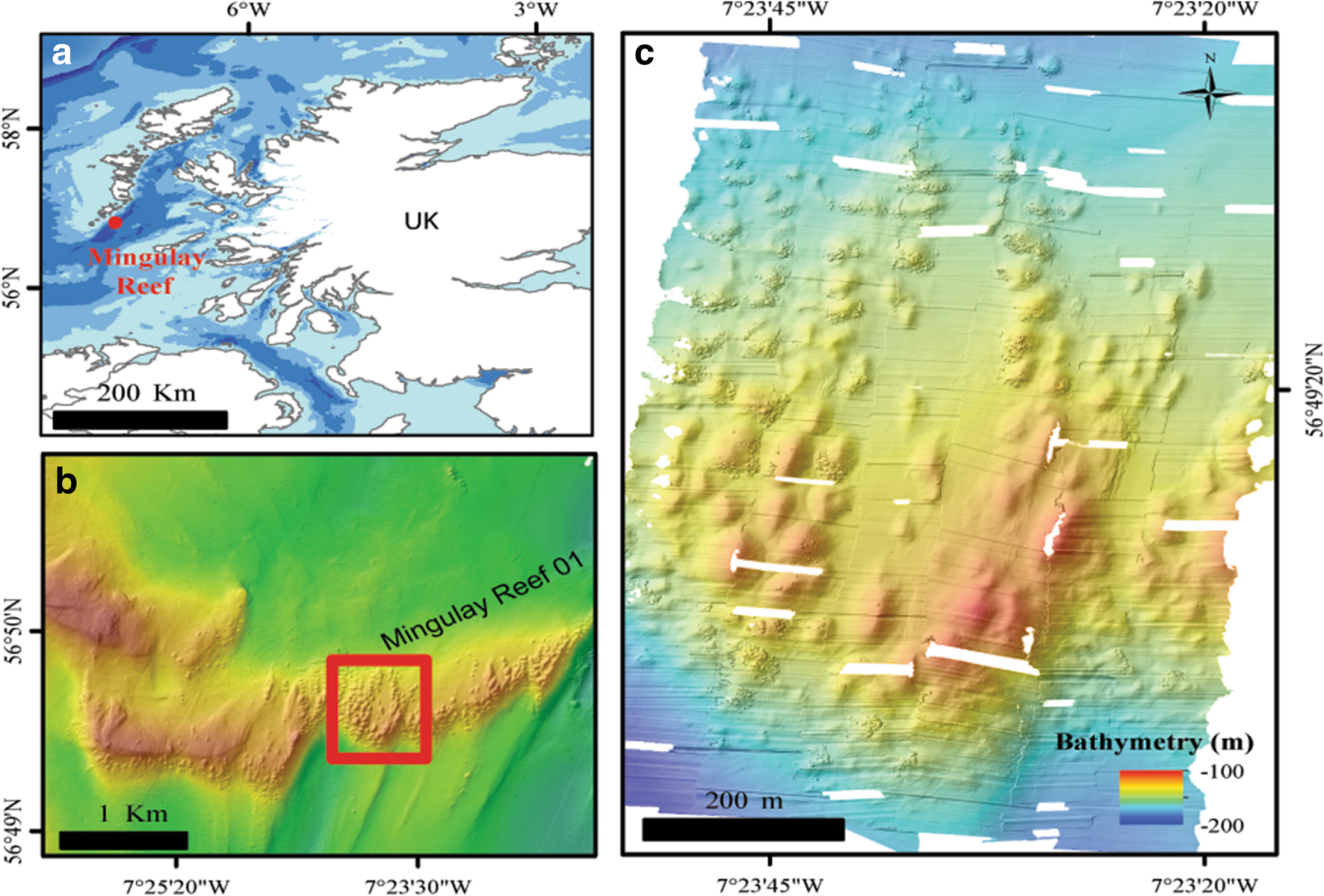
**a** Location of the Mingulay Reef Complex, **b** Mingulay Reef 01, **c** Microbathymetry area (location indicated by *red box* on panel **b**)

In this study, we focus on the Mingulay Reef Complex (MRC) (Fig. 1) located off the west coast of Scotland, where *L. pertusa* is the framework-forming coral. The MRC is a collection of several areas supporting reef mounds (Roberts et al. 2005). It is located in the Sea of Hebrides, south of the Little Minch in a passage between the Scottish mainland and the northern Inner and Outer Hebrides.

To date, predictive mapping of deep-sea organisms and habitats has mainly been based on video data, with or without grab or trawl samples (Roberts et al. 2009b; Henry et al. 2010; Howell et al. 2011). The advantage of video over sampling is that it covers large areas without disturbing the habitat. The exact position and local environment of the organisms are more uncertain for grab and trawl samples taken without precise in-water positioning, but they do provide higher detail and confidence when identifying species at lower taxonomic levels (De Clippele et al. 2015). A second drawback of biological sampling for predictive mapping is uncertainty regarding the absence of an organism in the whole area when it is not observed on the video. Even when large areas are covered with video surveys, the presence of a species that occurs outside the range of the camera’s view can be missed.

To overcome these drawbacks, a high-resolution microbathymetric grid (0.35 × 0.35 m) is used in this study. This grid covered a wider continuous area compared to a narrower, and discontinuous area covered by high-definition (HD) video transects. The microbathymetric grid allowed identification and mapping of individual coral colonies in their local spatial setting (Fig. 1c). HD video transects matching the colonies on the microbathymetry confirmed that the colonies were alive. Live coral presence and absence data were integrated from the microbathymetry with a broader resolution bathymetry (2 × 2 m) from which additional environmental variables were derived. Live coral presence data for areas outside the microbathymetry were included by using HD video transects.

Here, the focus lies on the east–west-oriented ridge of the MRC, Mingulay Reef 01 (MR1) (Fig. 1b), where we specifically studied live coral growth on the most extensive area of small *Lophelia* reef mounds in the MRC. Small reef mounds, referred to as ‘mini-mounds’, are widespread on MR1. In the spring and summer months, the water at the MRC is very turbid, caused by flocculent marine snow and some resuspension of existing seabed sediments (Fyfe et al. 1993), and the low visibility can interfere with the quality of video data. This near-seabed particle flux is caused by two mechanisms. The first is a rapid downwelling motion created during ebb and flood periods as an internal wave breaks over the largest of the five reef complexes, MR1 (Fig. 1b) (Davies et al. 2009). The ridge of MR1 is comprised of dolerite (Roberts et al. 2005). The ridge is relatively shallow with the shallowest point rising to less than 100 m water depth and the deepest point reaching 260 m. The hydraulic flow (or ‘jump’) can cause a depression of the downstream density structure which, when the tide reverses, is propagated back over the bank. This results in a supply of warmer, nutrient-rich water from the surface to MR1 (Davies et al. 2009). Organic matter resuspended from the seabed during peak tidal flows will be lower in quality but is a second mechanism of food supply.

A new British Geological Survey (BGS) seabed mapping toolbox was developed for the ArcGIS software to delineate the mini-mounds at MR1 semi-automatically from the broader-scale bathymetry data (2 × 2 m) and was used for the first time in this study. Cold-water corals occur at great depth, and therefore, the development of remote sensing approaches with semi-automatic systems is important. As multibeam bathymetry is commonly available for cold-water coral ecosystems, a potential wider application of this tool is possible. The characteristics of the mini-mounds that are delineated by the tool are extracted and integrated with live coral presence data from HD video material and from the microbathymetry. A map showing the prediction of live coral presence at MR1 will be useful for future long-term monitoring surveys, which can be used to gain insight in the impacts of global climate change.

## Methods

### Data

#### Bathymetry

Multibeam bathymetry data were collected at two different resolutions. One bathymetry data set was gathered using a Simrad EM2000 ship-mounted multibeam echosounder (MBES) as part of the MINCH project, using the RV *Lough Foyle* (28 June–5 July 2003) (Fig. 1b). This bathymetry covers the MR1 area and was processed to a resolution of 2 × 2 m. The system has an angular coverage sector of 120°, 111 beams per ping and a 1.5° beam width across the track. A Seapath 200 GPS system provided real-time heading, attitude, position and velocity solutions (Roberts et al. 2004).

The second bathymetry data set with a 0.35 × 0.35 m resolution (the microbathymetry) was acquired using the remotely operated vehicle (ROV) *Holland*-*1*, deployed from the RRS *James Cook* cruise 073 during the 2012 Changing Oceans Expedition (JC073, 18 May–15 June 2012) (Fig. 1c). A Reson 7125 dual-frequency multibeam echosounder system was mounted on the ROV and used in 400 kHz mode with 512 beams. Data were recorded in the Reson software PDS2000 and were supported by the inputs of the ROV depth sensor (Digiquartz, measuring depth in dbar), Motion Reference Unit (Phins), Doppler navigation and ultra-short baseline navigation (Sonardyne) (Roberts 2013). The microbathymetry covered only a small area (627 × 964 m) in the centre of MR1 (Fig. 1b, c). The navigation included the Doppler steering, but still showed a ‘saw-tooth’ pattern indicating a substantial number of sounding errors. This restricted the use of the map for calculation of additional derived environmental variables, but its high resolution still allowed us to use the map for visual interpretation.

Additional multibeam backscatter data were collected in 2012 (JC073) using the shipboard Simrad EM710 on the RRS *James Cook*. The grid has a resolution of 2.5 × 2.5 m (Le Bas and Huvenne 2009).

#### Current information

Maximum current speed was obtained from the hydrodynamic model of MRC created by Navas et al. (2014). The maximum current speed model has a lower resolution than the bathymetry and backscatter data (100 × 100 m) but provides local contextual environmental information that is not otherwise available.

#### HD video data

HD video data were collected in 2006 (64PE250 RV *Pelagia* cruise) and 2012 (JC073 RRS *James Cook* cruise). During the *Pelagia* cruise, two video monitoring systems were used: the Nederlands Instituut voor Onderzoek der Zee Hopper camera and the Scottish Association for Marine Science Bowtech umbilical video system. The first system consisted of a heavy drop frame holding a digital video camera with a logger, power supply, modem, UW lights, altimeter and a set of parallel Oktopus laser pointers. The laser pointers were spaced 30 cm apart and could be used to estimate the distance from the seafloor or the size of objects/organisms. The second system consisted of a much smaller frame and could be held 1–5 m above the sea floor by a winch. The system was also fitted with a Photosea strobe and camera unit and a set of parallel Oktopus lasers spaced 30 cm. Videos were captured to MiniDV tapes. Navigation data were collected with a Furuno DGPS system.

The second set of HD video data was collected using the ROV *Holland*-*1*. The data were recorded with a combination of cameras mounted on the ROV: an HD Insite mini-Zeus video camera with a direct HDSDI fibre output, a Kongsberg 14-208 digital still camera, a Kongsberg 14-366 pan and tilt camera and an Insite Pegasus-plus fixed zoom camera. Four lights were used: two 400-W deep-sea power and light SeaArc2 HMI lights and two 25,000 lm Cathx ocean APHOS LED lights. Two deep-sea power lasers spaced 10 cm were also mounted on the HD camera. The position and depth of the ROV were determined by USBL (Sonardyne) underwater positioning system and recorded using the OFOP (Ocean Floor Observation Protocol) software.

### Mapping tools

#### Semi-automated mapping: BGS seabed mapping toolbox

Although subjective manual mapping is the most common approach for identifying areas of potential coral habitats (Davies et al. 2008; Ross and Howell 2013), more objective and automated approaches have been published (Salcedo-Sanz et al. 2016). However, the semi-automated mapping approach presented in this study uses a fast and consistent automatic method and allows inclusion of additional data sets that have been developed within a GIS environment.

Development of the BGS seabed mapping toolbox for ArcGIS started after the successful creation of a semi-automatic workflow that can map and characterise pockmarks in the seabed (Gafeira et al. 2012). However, the approach had to be reversed to map positive features in areas of complex seabed morphology. The new workflow can be run as an ArcGIS tool. The first tool in the toolbox was named the BPI feature delineation tool. The script behind this tool follows a similar logic to the script developed for mapping pockmarks (Gafeira et al. 2012). The main difference is the use of the bathymetric positioning index (BPI) as an input instead of using bathymetric data directly.

The BPI uses neighbourhood analyses to calculate the relative elevation of a cell compared to the surrounding cells, identifying topographic features. Positive BPI values represent locations that are shallower than the average of their surroundings (e.g., ridges). Negative BPI values represent locations that are deeper than their surroundings. BPI values near zero are either flat areas or areas of constant slope (Weiss 2001). By using the BPI values as an input, the delineation will depend dramatically on how the BPI map was calculated. Here, the BPI was calculated within ArcGIS with the land facet corridor tool (Jenness et al. 2013). The used neighbourhood’s shape (annulus) and size (inner and outer radius of 8 and 24 cells, respectively) were chosen based on the characteristics of the study area (e.g., typical width of the mini-mounds, distance between features).

**Fig. 2:**
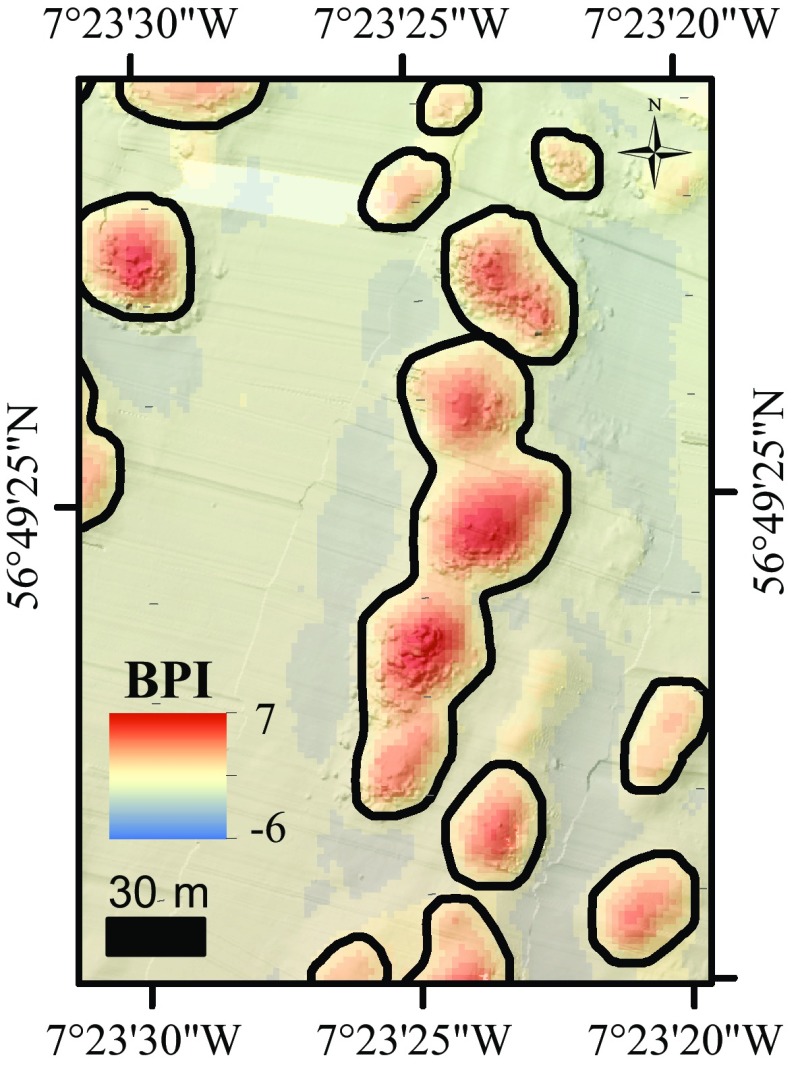
Bathymetric Positioning Index map (i8 × o24) with delineated mini-mounds

In the BPI feature delineation tool, several thresholds are decided by the user to determine the delineation process. These are the cutline BPI value, minimum BPI, minimum area and a minimum width/length ratio (Gafeira et al. 2015). Additionally, a buffer distance can also be defined within the tool dialogue box. This is applied to the polygons delineated to compensate for the fact that the delineation process was based on the cutline BPI value, which normally reaches the mound’s lateral slopes rather than its base. The tool output is a polygon shapefile delineating the areas of seabed respecting the dimensions and BPI values set by the user. A cutline BPI value of 3, minimum BPI value of 3.5, minimum area of 50 m^2^ and a minimum width/length ratio of 0.2 were used as the thresholds. A buffer distance of 4 m was chosen based on the morphological characteristics of the mounds and how they are represented in the BPI raster used as data input. Nevertheless, some degree of manual editing was required when adjacent mini-mounds were delineated as a single feature (Fig. 2). More detailed information regarding the BPI feature delineation tool and the results of the sensitivity to the BPI input and threshold values can be found in Gafeira et al. (2015).

**Fig. 3:**
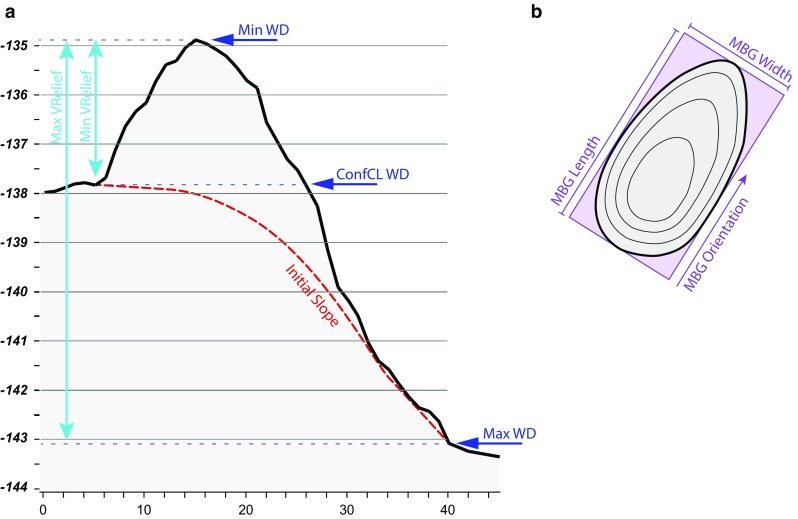
**a** A bathymetric profile of a mini-mound at the edge of the Mingulay Reef Complex, showing the position of the minimum water depth (Min WD), water depth of the deepest confined contour line (ConfCl WD) and maximum water depth (Max WD), plus the vertical relief correspondent to maximum (Max VRelief) and minimum vertical relief (Min VRelief). **b** Minimum bounding geometry box length (MBG length), width (MBG width) and orientation (MBG orientation) of a polygon delineating a mini-mound

The table of attributes of the output shapefile of the BPI feature delineation tool captures some characteristics of the mapped features (e.g. area, perimeter, maximum BPI value and minimum bounding geometry box length). However, after manually editing the outline of some of the polygons the information on it could be partially incorrect. The second tool in the toolbox, the feature description tool, recalculates these attribute values for each feature before adding new attribute fields with additional information. At this point of the toolbox development, the feature description tool allows extraction of information from the original digital elevation model (DEM), the BPI map, backscatter and rugosity map. Backscatter data give information on the physical attributes of the seabed (Todd et al. 1999; Kostylev et al. 2001). High values tend to represent substrates that are more reflective, such as boulders or rocks. Lower values represent environments where reflection by the substrate is lower, such as muddy areas. Rugosity was calculated as the standard deviation of the slope in ArcGIS. It is a measure of terrain roughness and is obtained as the ratio of the 3D surface area to the planar area within a neighbourhood (3 × 3 cells) (Jenness 2002). This tool can also calculate the assumed initial slope of the seabed surface before the development of the coral mound. The initial slope is calculated by removing the bathymetric information from inside the delineated mini-mounds and generating a new surface by interpolating from the surrounding bathymetric values. The mean value of the assumed initial slope within each mini-mound is extracted and added to the table attributes. This tool can extract 20 descriptive attributes (Table 1). Of these, minimum/maximum vertical relief, minimum/mean/maximum water depth, water depth of the deepest confined contour line, width, length and orientation are illustrated in Fig. 3. Current data were not extracted automatically by the tool but added later through the joins and relates function in ArcGIS.

**Table 1:** Variables calculated by the feature description tool

Variable	Meaning
**Area**	**Area in square meters**
Perimeter	Length of the polygon boundary in meters
**Index (BPI)**	**Maximum bathymetric positioning index**
**MBG_Width (m)**	**Minimum bounding geometry box width**
**MBG_Length (m)**	**Minimum bounding geometry box length**
**MBG_W_L**	**MBG_Width/MBG_Length**
**MBG_Orient**	**Minimum bounding geometry box orientation**
MinWD	Minimum water depth in meters
**MaxWD**	**Maximum water depth in meters**
MeanWD	Mean water depth in meters
ConfCL_WD	Water depth of the deepest confined contour line
**MinVRelief**	**Minimum vertical relief in meters**
**MaxVRelief**	**Maximum vertical relief in meters**
**Min_Rug**	**Minimum rugosity value**
**Max_Rug**	**Maximum rugosity value**
**Mean_Rug**	**Mean rugosity value**
**Min_BS**	**Minimum backscatter value**
**Max_BS**	**Maximum backscatter value**
**Mean_BS**	**Mean backscatter value**
**InitialSlp**	**Initial slope in decimal degrees**

The variables in *bold* were used in the model. Maximum current speed is not listed here as it was not derived trough the feature description tool

#### Coral presence detected using microbathymetry

**Fig. 4:**
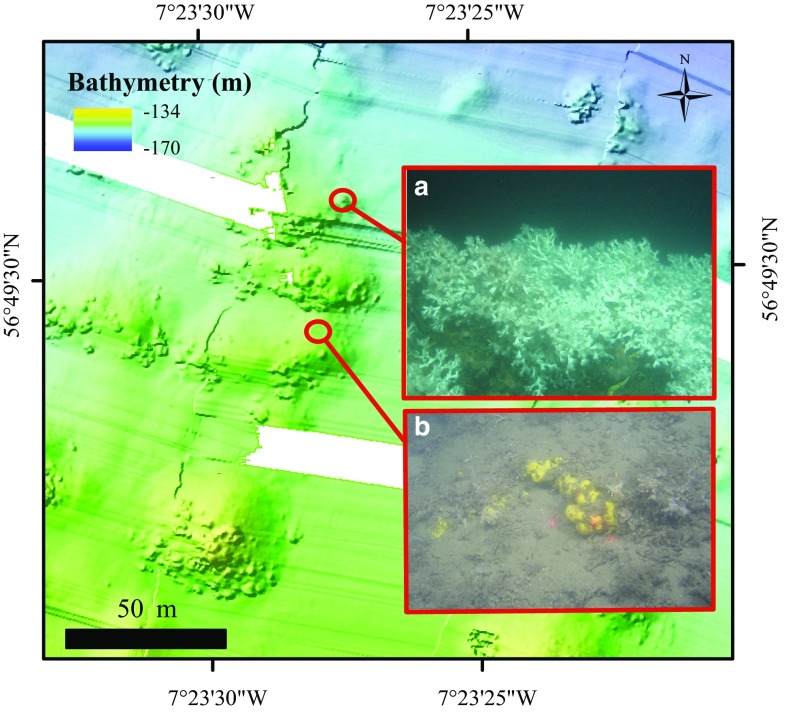
Detail of microbathymetry illustrating **a** a live coral colony and **b** the substrate found in-between mini-mounds (rubble, fine sediment, sponges and *Parazoanthus anguicomus*)

**Fig. 5:**
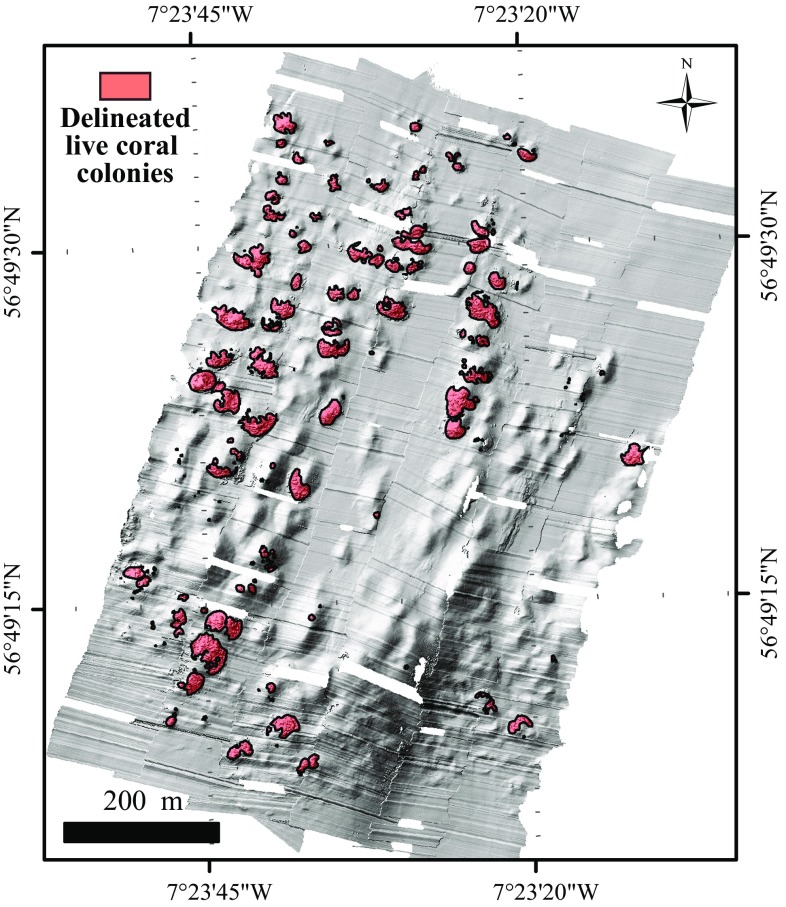
Microbathymetry indicating areas covered with coral (*red shading*)

**Fig. 6:**
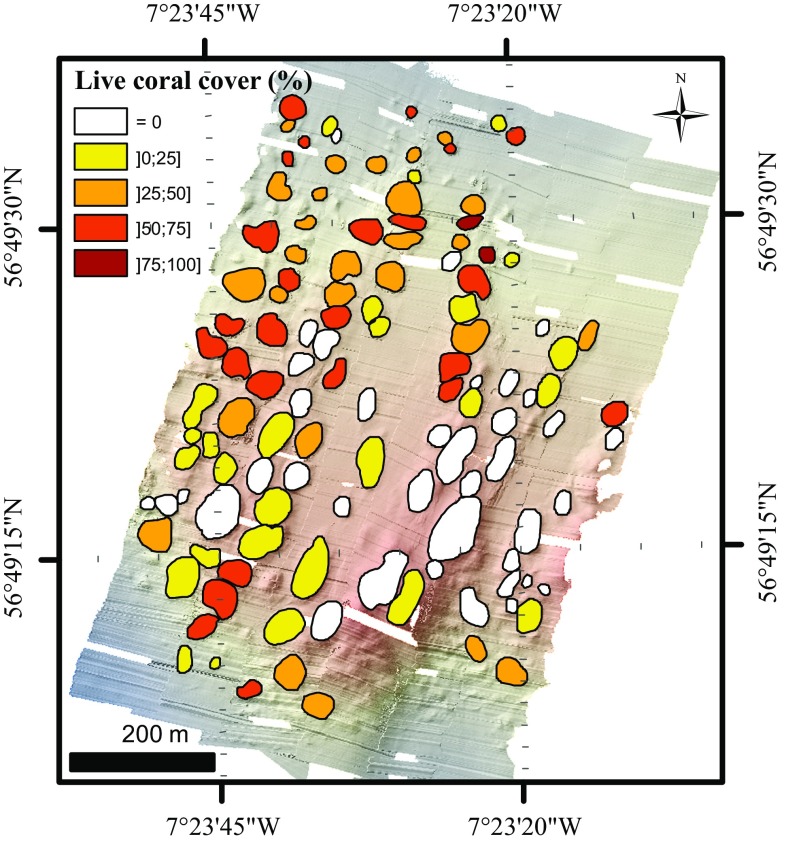
Distribution of mini-mounds in five classes of percentage live coral cover (= 0, ]0;25], ]25;50], ]50;75] and ]75;100]) in the microbathymetry area

The microbathymetry showed small circular features and irregularities on the mini-mounds that were identified with HD video transects as live coral colonies (1–7 m in diameter) (Fig. 4). Their distribution and extent were mapped within ArcGIS (through manual delineation; Fig. 5), which allowed calculation of the percentage of live coral cover per mini-mound. Mini-mounds were divided into five different classes based on coral cover: = 0, ]0;25], ]25;50], ]50;75], ]75;100] (Fig. 6).

### Spatial distribution modelling

#### Random forest classification

Random forest (RF) classification (Breiman 2001) is used here as a modelling technique. A random forest consists of a number of simple decision trees. Each tree is based on a bootstrapped sample of the biological and environmental data set. This group of simple trees vote for the most popular class, which is capable of predicting a response when presented with a set of explanatory variables. More background information about the application can be found in Cutler et al. (2007) and Rogan et al. (2008). All classifications were carried out in R version 3.2.2 (R Development Core Team 2013) using the random forest package v.1.6-7 (Liaw and Wiener 2002). Here, the mini-mounds as a whole are used as samples. Three different models were compared. In the first model, the percentage of live coral cover on the mounds was modelled in the five different classes (120 samples). In the second model, we modelled the presence/absence of live corals on the mounds within the microbathymetry (120 samples). In the third model, the presence of live coral samples from the HD video analyses was included in the presence/absence model (132 samples). The RF classification models were assessed based on random separation of training and test (validation) data. This was done by leaving one-third of the data out for model training and subsequently using them to test the model. The out-of-bag (OOB) error gives an indication of the prediction error of RF and is calculated based on the bootstrapped samples (Breiman 2001).

#### Predictor variables and their importance within the models

The 20 descriptive variables extracted using the feature description tool plus the current value of each delineated mound were considered as predictor variables for the RF models. Initially, the models were estimated by using all the variables. A plot was created with the mean decrease in accuracy for each variable indicating its contribution to the model’s performance. Response curves were calculated to show the range of values for each variable for which there is the highest chance of presence.

#### Evaluation of the model

Sensitivity and specificity were calculated for both the training and test models. Sensitivity measures the proportion of positives that are correctly identified, whereas specificity measures the proportion of negatives that are correctly identified. The discrimination capacity of the model was assessed using the area under the receiver operation curve (AUC), an evaluation metric for binary classification problems. The AUC value for a model can indicate poor (<0.5), random (0.5–0.6) fair (0.7–0.8), good (0.8–0.9) or excellent (0.9–1) discrimination (Fielding and Bell 1997). Higher AUC values for the test models indicate that the training model is good at discriminating between samples or classes.

## Results

### *Lophelia* reef mini-mounds and presence of living coral framework

**Fig. 7:**
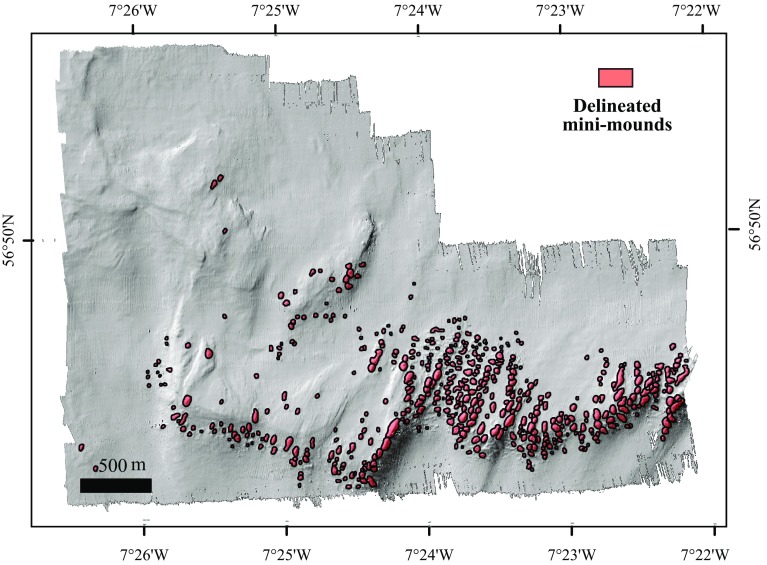
Mingulay Reef 01 with mini-mounds delineated in *red*, obtained with the BPI delineation tool

A total of 505 mini-mounds were delineated by the BGS seabed mapping toolbox in the whole MR1 area (Fig. 7). The mini-mounds were between 13 and 60 m wide and 16–108 m long, with an area of 174–4645 m^2^.

Of 120 mini-mounds mapped in the area of the microbathymetry data set, 82 mini-mounds were covered with circular features 1–7 m in diameter, indicating the presence of live coral framework as confirmed with HD video records (Fig. 4). The remaining 38 mini-mounds showed no signs of live corals of this size. We calculated that 6% of the microbathymetry area was covered with live coral. The remaining 94% was a mixture of coral rubble covered with the zoanthid *Parazoanthus anguicomus* and sponges, together with fine sediment (Fig. 4). Using the videos from 2006 and 2012, living coral colonies were identified in 29 of the 124 mini-mounds surveyed. Of these 29 mini-mounds, only 12 were outside the microbathymetry area. Seven of these were situated to the east of the microbathymetry area, and five to the west.

Visual interpretation showed that the corals were more frequently located on the southwest side of the mini-mounds, facing into the residual current flow to the northeast. Corals seemed to be growing both directly on the mini-mounds and at their base. Corals that were associated with mounds covered between 2 and 1417 m^2^. Coral colonies that were not associated with mini-mounds were much smaller and covered surface areas from 0.3 to 23 m^2^. In the HD video samples, no absence data are included as true absences could not be determined based on a partial survey on the mounds.

### Random forest classification

Table 2 displays the results of the RF classification for the three different models. The out-of-bag (OOB) error rate for the percentage cover model was the worst, with a value of 42.5% (Table 2a). This high error rate is caused by the greater number of categories that can be mis-classified. The scenario presence/absence model had an error rate of 21.95% (Table 2b). The scenario including the HD video samples had the lowest error rate of 18.18% (Table 2c). These OOB values are given for models after the removal of the correlated environmental variables (see section ‘variable selection’). The last scenario was further adapted to create a predictive map.

**Table 2:** Random forest classification training model results for (a) Percentage live coral cover, out-of-bag (OOB) estimate of error rate: 42.5%, (b) presence (1)/absence (0) of live coral cover within the microbathymetry, OOB estimate of error rate: 21.95% (c) presence (1)/absence (0) of live coral cover within the microbathymetry with high-definition samples, OOB estimate of error rate: 18.18%

(a)	Percentage coral cover					Class.error
	=0	]0;25]	]25;50]	]50;75]	]75;100]	
=0	19	4	1	2	0	0.269
]0;25]	5	10	4	2	0	0.524
]25;50]	0	2	8	5	0	0.466
]50;75]	1	3	4	9	0	0.470
]75;100]	0	1	0	0	0	1.000

(b)	1	0	Class.error			
1	49	5	0.070			
0	14	12	0.560			

(c)	1	0	Class.error			
1	58	5	0.079			
0	11	14	0.440			

Number of trees = 1500, number of variables per level = 6

#### Variable selection

**Fig. 8:**
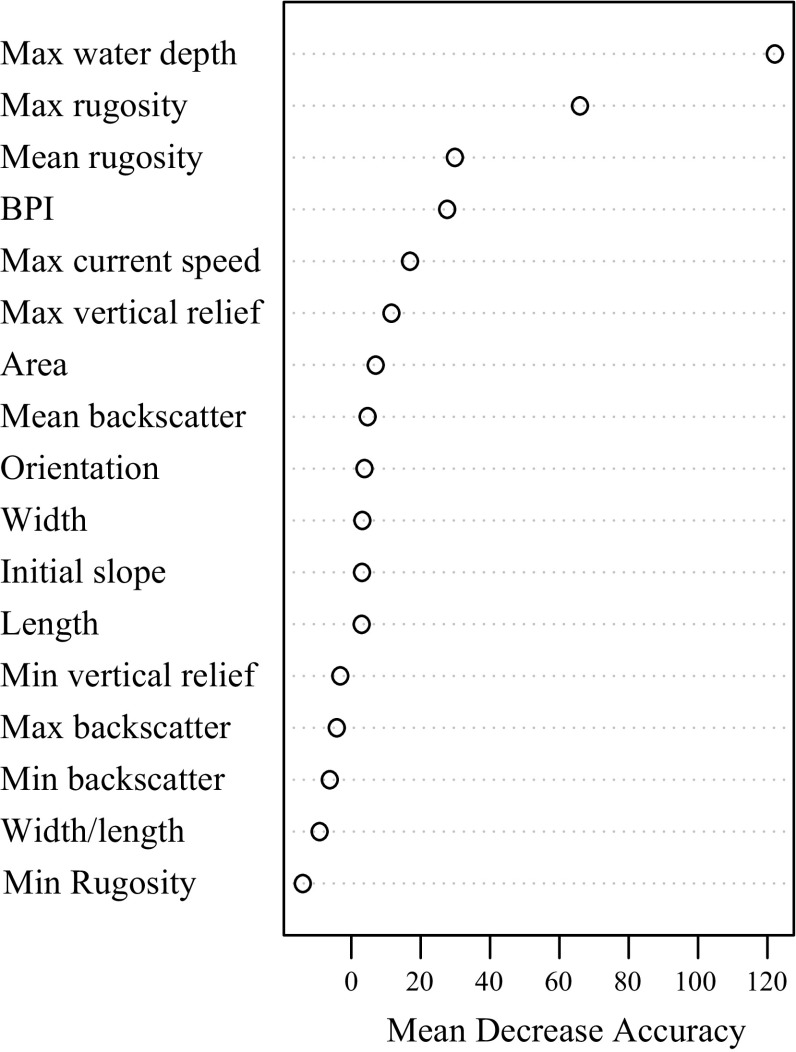
Mean decrease in accuracy plot indicating the contribution of each variable to the model performance

**Fig. 9:**
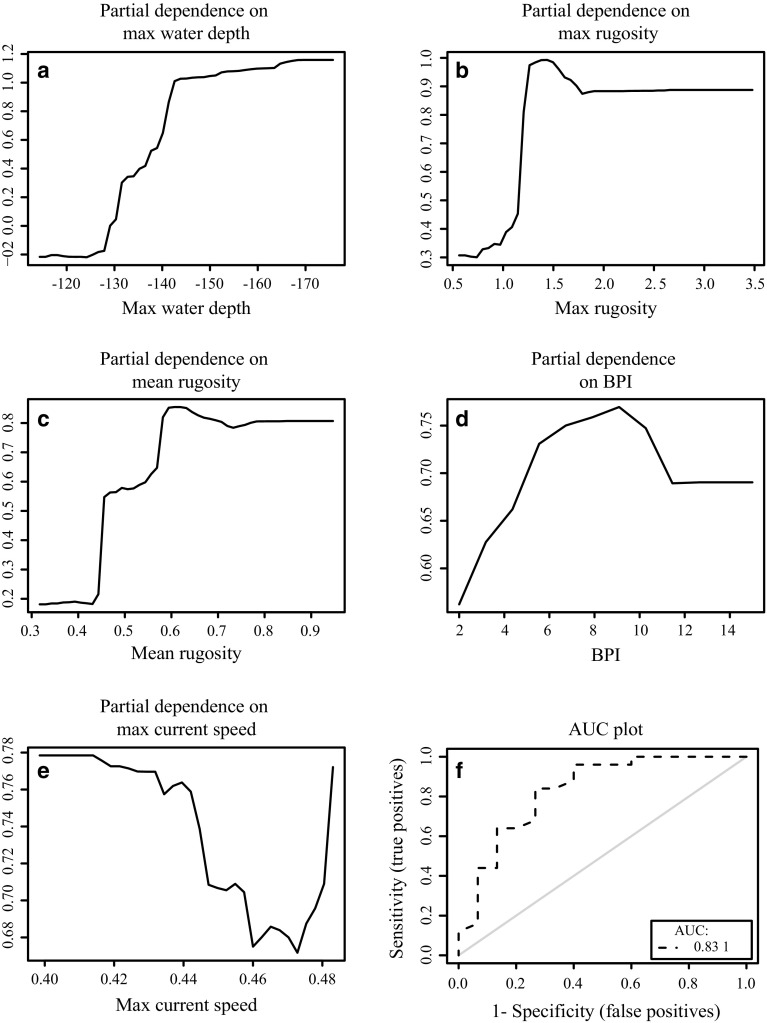
Response curves for the partial dependence on **a** maximum water depth, **b** maximum rugosity, **c** mean rugosity, **d** bathymetric positioning index and **e** maximum current speed. These are the variables that contributed the most to the performance of the model chosen for prediction. These response curves give the partial probability for live coral presence on the delineated mini-mounds as a function of the individual predictor variables **f** With an AUC value of 0.83, this plot shows that the test model of the ‘presence/absence data from the microbathymetry area + presence data from the HD video samples’, is good at discriminating between samples

The water depth of the deepest confined contour line and the minimum, mean and maximum water depth were highly correlated (*r* ^2^ > 0.95). Therefore, only maximum water depth was included, as this variable explained the most variation in the data. The maximum water depth, maximum rugosity, BPI, orientation and maximum current speed were the five most important environmental contributors to the model, as indicated by the mean decrease in accuracy for each variable (Fig. 8). The response curves (Fig. 9a–e) illustrate the range of values of the predictor variables and the associated partial probability of live coral presence.

#### Model validation

**Fig. 10:**
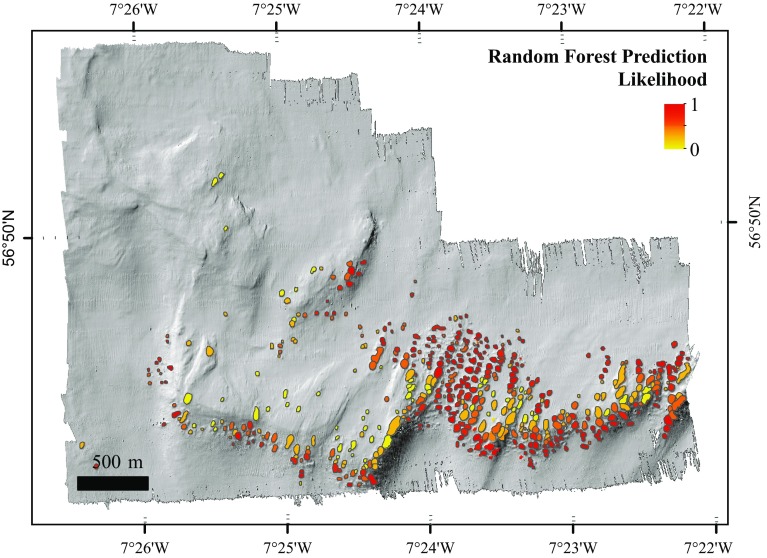
Random forest modelled distribution showing the likelihood of live coral presence on the delineated mini-mounds in the Mingulay Reef Complex. *Lighter colours* represent a higher likelihood of absence, while *darker colours* represent a higher likelihood of presence of live coral colonies

The sensitivity for the training data of the HD video samples model was 0.92, and the specificity was 0.56. The higher sensitivity indicates that the model was better at predicting presence than absence. The AUC value for the test data was 0.83 (Fig. 9f), indicating that the model performed very well and was therefore used to predict the probability of the presence of live corals on mini-mounds in the wider MR1 (Fig. 10).

#### Predictive map

The predictive map (Fig. 10) illustrates that the highest probability of finding mini-mounds with live coral was at the centre of the reef, near the channel. The north side of the eastern part of the reef had more mini-mounds together with a high probability of the presence of live coral. The western part of the reef had lower coverage of mapped mini-mounds, with low probability of live coral on the south side and higher probability on the north-facing side. A smaller ridge-shaped feature located north of the reef also has a high probability of live coral, especially along the north and eastern sides.

## Discussion

This is the first study to map living cold-water coral colonies acoustically through an ROV-based microbathymetric grid and the newly developed BGS seabed mapping toolbox. This allowed us to explore the environmental variables that control coral growth on mini-mounds at MR1. The microbathymetry provided very clear morphological evidence for the presence of live coral colonies and allowed us to calculate the percentage living coral cover on the mini-mounds of MR1. The BGS seabed mapping toolbox was an efficient and reliable tool to identify these mini-mounds rapidly and to calculate a variety of environmental factors for each mini-mound. By integrating these data sets, a quick assessment of the morphology of the mini-mounds and the environmental factors that determine which mini-mounds support or do not support live coral is possible. Here, the strongest explanatory model was computed for coral presence/absence with HD video samples.

### Environmental variables

#### Maximum water depth and current speed

Maximum water depth was the most important contributor to coral presence. The microbathymetry showed that mini-mounds without live coral were more abundant on the upper part of the ridge, while mini-mounds with live corals were more likely to be found at the edges of the ridge of MR1 (Figs. 5, 6, 10). The proximity of the mini-mounds to the surface could be a factor contributing to coral absence. Mingulay Reef is mainly supplied with food from surface waters through tidal downwelling, which changes environmental conditions experienced by the corals semi-diurnally (Davies et al. 2009; Findlay et al. 2013). The range of environmental changes is not consistent with depth (Findlay et al. 2013), which could contribute to the absence of corals. Corals at shallower depths are also more likely to be exposed to higher turbulence caused by higher current speeds. This could cause the polyps to retract to avoid damage, thus reducing feeding compared to deeper corals (Sebens and Johnson 1991; Dai and Lin 1993; Purser et al. 2010).

Maximum current speed was slightly lower for mini-mounds with live corals than for mounds without live coral cover. This may be related to less optimal conditions at the top of the ridge. Davies et al. (2009) measured current speeds at the top and foot of MR1 and found maximum current speed of 26 cm s^−1^ at the foot but maximum current speeds of 81 cm s^−1^ (east) and 67 cm s^−1^ (west) at the top. At greater depths, current speed, temperature and turbulence will be lower. However, high current speeds may not be optimal for coral feeding. When the currents are too low, corals are limited by the amount of food passing the polyps. When the currents are too high, polyp tentacles are bent backwards, reducing their ability to capture food (Sebens and Johnson 1991; Dai and Lin 1993; Purser et al. 2010). In slower currents, corals may also be more successful in bringing food to their mouth, as prey could break loose more easily under higher flow conditions that provide more momentum to escape from the entrapping mucus strands of the tentacles (Patterson 1984; Tsounis et al. 2010). It is important to note that the differences in depth and current speed between mini-mounds with and without live coral cover are small. Fine-scale nutrient and current data are needed to investigate this pattern with more certainty.

#### Rugosity and bathymetric positioning index

Rugosity is the second-most important variable related to coral presence. This is a variable that is more related to the morphological properties of the mini-mound and is therefore not an independent characteristic of the mini-mound. Mini-mounds with high live coral cover are more likely to have greater maximum and mean rugosity than mini-mounds with only dead corals. When the growth of corals increases, the unevenness of the mound structure increases as the coral polyps grow outwards to obtain better passing food. Mini-mounds composed of rubble without live coral, covered by *Parazoanthus anguicomus* and sediment (Fig. 4), have lower rugosity as there is less morphological complexity (observation from HD videos). Erosional processes will also reduce the unevenness of the mound and of the reef structure, as when the corals die, the underlying framework may be rapidly bioeroded until it collapses. Rugosity is easily calculated from bathymetry and can, therefore, be used to identify the potential occurrence of cold-water corals in other areas.

As anticipated, BPI was higher for mini-mounds with living coral colonies. Corals are typically associated with areas of accelerated near-bed currents which are often found on sloping topographies and topographic highs because the encounter rate of food increases with higher current speeds (Genin et al. 1986; Frederiksen et al. 1992 Mortensen 2001; Thiem et al. 2006). Coral colonies can therefore be found on mini-mounds with a higher BPI as these mounds are more exposed to these preferential faster current speeds. At MR1, the formation of mini-mounds is possible as the tidal currents create a highly energetic environment with favourable food and temperature conditions. In deeper offshore settings, coral carbonate mounds can be several hundred metres high and can, therefore, be picked up by BPI on coarser bathymetric grids (Wilson et al. 2007). This feature also forms the basis for the use of the BGS seabed mapping toolbox. The summits and upper flanks of these coral carbonate mounds are highly diverse and are capped by living cold-water coral and a rich associated community, consisting of sponges, crinoids and crustaceans.

### Interpretation of predictive map

The Mingulay *Lophelia* reefs at MR1 have developed primarily to the centre and to the south of the west–east-trending ridge which rises to 40–80 m above the surrounding seabed (180–200 m depth). This can be explained by higher current speeds passing the centre of the reef through a channel and with the south side of the ridge lying perpendicular to the current. The ridge structure is an obstruction to the tidal flow and so modifies the strength and direction of the near-bed flow. The north side of the ridge is more sheltered and therefore less preferred by the corals. Currents in the area are largely tidal, and their strength is altered by the topography. On the eastern part, current amplitude reached 29 cm s^−1^ with a peak of 81 cm s^−1^. The western part is more sheltered from the tidal currents with current speeds of 22–67 cm s^−1^ (Davies et al. 2009). The higher abundance of live coral in these locations could also be related to the temperature–depth relationship; the corals in the centre and on the north side of the ridge will be exposed to lower temperatures and therefore be more likely to survive.

### Advantage of HD video records

The model that included the sample data from the microbathymetry area and the HD video data performed the best. This model was better at predicting presence of live coral colonies than predicting their absence. This was expected, as there was no absence data available from outside the central microbathymetry area. If microbathymetry data covering a wider area of the reef were available, true absence data could be included which would make the model more accurate. This would result in a more robust model for both presence and absence. Predictive models are sensitive to the number of presence points incorporated into the analyses but the success differs among approaches (Stockwell and Peterson 2002; Bryan and Metaxas 2007). Adding more presence data on corals from outside the microbathymetry area gave a more equally distributed data set accounting for the variation in environmental variables across the reef. This study highlights the advantage of using microbathymetry, which allowed us to observe real presence and absence complemented with HD video data to identify live coral colonies. However, a wider coverage of microbathymetry would allow better coverage of both presence and absence of live coral over the range of environmental variables. Combining the centrally located microbathymetry with HD videos gave robust results for presence but less robust results for absence of live coral colonies.

### Future implications: climate change

Our understanding of the small-scale local variation of coral growth at MR1 can be applied to monitor future changes, from anthropogenic impacts (e.g., bottom trawl damage) to the impacts of global change including ocean warming and acidification. Corals at the Mingulay Reef Complex are subjected to daily tidal downwellings. During the summer stratified period, corals on the shallowest parts of the reef are exposed to warmer water of higher pH that is nutrient-depleted but rich in phytoplankton-derived particles compared to corals located at greater depths. If summer temperatures increase over time due to climate warming, the downwelling over Mingulay Reef could increase stress on the cold-water corals (Findlay et al. 2013). The interplay between current speeds, food supply and temperature is key for coral presence at the MRC, and any changes could result in a smaller optimal growth range at the MRC and other reefs that thrive in similar conditions.

Ocean acidification is of major concern for many marine ecosystems, particularly calcifiers already growing in water masses close to carbonate under-saturation such as cold-water coral reefs (Guinotte et al. 2006; Roberts et al. 2006; Turley et al. 2006; Aze et al. 2014; Hennige et al. 2014). Ocean acidification impacts not only surface waters but the carbonate chemistry at depth (Caldeira and Wickett 2003). Studies on ocean acidification suggest that ocean pH will decrease by approximate 0.3–0.4 pH units by the end of the century (Guinotte et al. 2006; Roberts et al. 2006; Turley et al. 2006). This will also influence the depth of the aragonite saturation horizon (ASH), which creates conditions that are less favourable for skeletal growth (Orr et al. 2005; Guinotte et al. 2006; Turley et al. 2007). However, the MRC is unlikely to be affected by the shoaling of the ASH. This makes the reef a possible site of interest as a refuge for cold-water corals in a future ocean where ocean acidification has negatively impacted deeper corals. For the MRC seasonal surface uptake of food, coastal run-off and increasing seawater temperatures will be of higher importance than the effects of ocean acidification (Findlay et al. 2013). Findlay et al. (2013) showed that cold-water coral reefs like the MRC may be subjected to a more variable biogeochemical environment than previously thought. Hennige et al. (2015) showed that *L. pertusa* can adapt to a combination of increasing CO_2_ and temperatures at the same time. However, this adaptation came with a trade-off for the physical strength of the coral. Coral colonies will, therefore, be likely to be more affected by bioerosion and physical damage by 2100.

Our detailed map of the current presence of live corals on mini-mounds can function as a baseline and tool for future sampling and monitoring. Even though the bathymetry and video data were collected over several years, the impact on our results would be minimal. Growth rates of *L. pertusa* vary widely, ranging from 2.6 to 20–25 mm yr^−1^. Even if corals grew at their maximum growth rate of 25 mm yr^−1^ from 2003 to 2012, the corals would have only grown 225 mm. After testing the model by ground-truthing, this approach could be used to guide sampling towards mounds with the highest probability of coral presence to maximise ship time.

Present and past small-scale hydrodynamic data could shed light on the processes that shaped the current distribution of the mini-mounds on the MRC. Long-term temperature measurements are lacking; by obtaining this data, the sensitivity of the MRC to climate warming could be explored in more detail. We show that live coral colonies can be identified from a microbathymetry map. Microbathymetry maps of other parts of the reef can, therefore, be useful for further monitoring and can be used to ground-truth our results. Our study highlights the need for fine-scale environmental data, as small-scale processes control live coral presence on the mini-mounds at the MRC.
